# A Pilot Study of Nuclear Instability in Archived Renal and Upper Urinary Tract Tumours with Putative Ochratoxin Aetiology

**DOI:** 10.3390/toxins2030326

**Published:** 2010-03-09

**Authors:** Peter G. Mantle, Cyrille Amerasinghe, Amy L. Brown, Diana Herman, Thomas Horn, Thoger Krogh, Edward W. Odell, Tomas Rosenbaum, Calin A. Tatu

**Affiliations:** 1 Centre for Environmental Policy, Imperial College London, London SW7 2AZ, UK; 2 Department of Histopathology, Ealing Hospital, Southall, Middlesex, UB1 3HW, UK; 3 Department of Oral Pathology, Kings College London, London, UK; 4 Pathology Department, County Hospital Timisoara, Romania; 5 Pathology Department, Herlev Hospital, University of Copenhagen, DK-2730 Herlev, Denmark; 6 Department of Rheumatology, Aarhus University Hospital, 8000 Aarhus. Denmark; 7 Department of Urology, Ealing Hospital, Southall, Middlesex. UB1 3HW, UK; 8 Department of Biology, University of Medicine and Pharmacy, Timisoara, Romania

**Keywords:** DNA ploidy, Balkan endemic nephropathy, renal cell carcinoma, ochratoxin A, porcine nephropathy, metastasis, nuclear instability, transitional cell carcinoma, aneuploidisation

## Abstract

DNA ploidy measurement has been applied uniquely to wax-embedded tissue of primary renal cell and metastatic tumours of a key experimental researcher on porcine ochratoxicosis, a control, and four transitional cell carcinomas from cases of Balkan endemic nephropathy. Primary renal tumour was diploid, and hyperdiploid metastasis was within the lower ploidy range for typical renal cell carcinoma. Three Balkan primary tumours showed extensive aneuploidy indicating marked nuclear instability, similar to model rat renal carcinoma caused by ochratoxin A. In contrast, much less nuclear instability in the putative occupational ochratoxicosis case fitted poorly with the ochratoxin A model.

## 1. Introduction

The study arises from the potent nephrocarcinogenicity of the mycotoxin ochratoxin A (OTA), the fact that most human renal tumours are idiopathic, and the uncertain designation of dietary OTA as a human health risk.

Ochratoxin A is one of the first-discovered mycotoxins. Recognised in South Africa as a fungal metabolite that was toxic to experimental rats [1], the structure was also determined there [2]. OTA became more widely important in the 1970s when implicated in the aetiology of the chronic porcine nephropathy that was occurring sporadically in Danish pigs [3,4]. OTA occurrence in Denmark was associated particularly with mould spoilage of home-grown cereals destined for pig feed that had not been dried adequately before storage.

A curious human nephropathy was recognised as a discrete entity in the Vratza district of Bulgaria in the mid-1950s [5,6] and gradually became recognised more widely within the Balkans as an idiopathic nosologic entity of mosaic occurrence in certain households in certain agricultural villages. It manifests as a silent progressive bilateral renal atrophy that generally becomes apparent in late middle age. The pathology was addressed formally by the World Health Organisation [7], was a topic for a CIBA Foundation Study [8], and articles on it gradually appeared in Western European literature, summarised in [9]. Concurrently, there was a climate of expanding discovery of fungal metabolites (e.g., aflatoxin B1, zearalenone and several trichothecenes) as potent agents of natural mammalian disease. Since Danish veterinarian Professor Palle Krogh had been much involved in large-scale pig experiments on OTA and renal disease, it was just imaginative lateral-thinking for him to propose that the mycotoxin might also be an agent of human disease, particularly the Balkan endemic nephropathy [10]. Balkan endemic nephropathy (BEN) occurred mainly in flood-plain regions associated with the Danube and its tributaries, but was focused on particular households, irrespective of ethnic origin, in rural communities in which storage conditions for agricultural commodities, predominantly home-grown for humans and for pigs, were susceptible to mould spoilage. Thus causal association with Danish porcine nephropathy was readily made [3] following detection of OTA in cereals [11] and experimental studies based on the natural occurrence of OTA in Danish cereals [12]. Consequently, research was stimulated within and outside Balkan countries into the human disease, the extent and identity of endemic mould sources of OTA, the incidence of the toxin in human diet, and on assessing the potential human risk of traces of OTA in human food.

Whereas, in its original South African discovery, OTA was a metabolite of an isolate of *Aspergillus ochraceus,* which is not a part of the natural mycobiota in Northern European latitudes (where *Penicillium verrucosum* was the producing fungus), it was not easy to predict the source in the Balkans, where climate is more temperate although winters can be cold. 

Studies on moulds contaminating foodstuffs from nephropathy households in hyperendemic villages in Croatia and Bulgaria revealed no evidence of ochratoxinogenic *P. verrucosum*; *A. ochraceus* was very rare and usually was not ochratoxinogenic in laboratory culture on cereal substrate [13,14]. Concurrently, *A. ochraceus* was isolated from 10% of 855 samples of stored cereals and dried meats collected (1980-1987) from nephropathy and non-nephropathy households in Croatia [15]. One third of isolates were slightly ochratoxinogenic in laboratory culture but only one, from ham, was a prominent producer with yield similar to a reference culture. It was concluded that there was no significant difference in ochratoxinogenic mycobiota between the two regions. Otherwise, the only other systematic mycological study was on 14 samples of poor quality wheat tailings used in feed of pigs expressing typical clinical symptoms of mycotoxic porcine nephropathy in Bulgaria [16]; *A. ochraceus* was a rare component in only four of these but was not ochratoxinogenic in laboratory culture.

The problem of mycotoxic porcine nephropathy in Denmark became less prevalent as moisture in grain for storage became controlled more rigorously. Consequently, concern over OTA waned somewhat until results of a US National Toxicology Programme (NTP) study on the toxicology of OTA appeared [17], showing it to be the most potent rat renal carcinogen found to date. This distinction still prevails [18]. Additionally, the incidence of upper urinary tract tumours amongst subjects diagnosed with the Balkan nephropathy was found to be up to 100-fold higher than amongst Balkan populations not affected by the bilateral renal atrophy [19]. Naturally, concern then became heightened regarding a putative added aetiological connection between OTA and human urological tumours, most of which were, and still are, of uncertain/unknown cause.

Shortly before the NTP study was published, Palle Krogh was diagnosed with a renal cell carcinoma (RCC) requiring radical nephrectomy in 1988, but tragically he died 2 years later. This might ironically have been a unique case of potential occupational accidental exposure to the potent OTA during his practical involvement in large-scale pig experiments before its carcinogenic potential had been fully realised. To our knowledge, no person who has died from renal cell carcinoma has also been shown specifically to have been exposed to abnormal amounts of OTA. Trace amounts (~1 ppb) of OTA are found widely in human blood but no human morbidity has yet been attributed conclusively to OTA.

Recently, the opportunity arose to measure DNA ploidy distribution in experimental rat renal tumours that had occurred in response to dietary OTA during lifetime studies [20] and associated experiments]. Differentiation of tumours into adenomas and carcinomas by histopathological criteria was consistently correlated with diploid and markedly aneuploid status, respectively [21]. That study has now been extended to include some archived human pathological material, particularly including primary renal tumour and a distant metastatic tumour of Palle Krogh, and a control renal tumour for which abnormal OTA exposure was not suspected. The technique has been extended into a pilot study of four archived upper urinary tract transitional cell carcinomas (TCC) from cases of Balkan endemic nephropathy to test its efficacy and contribute to current debate on the validity of extrapolation from animal experiments with OTA to human risk. The pilot study also assessed potential for meaningful application of DNA ploidy measurement to the extensive histological archives of TCC from Balkan nephropathy cases, as well as to currently-emerging cases. 

## 2. Results

**Figure 1:**
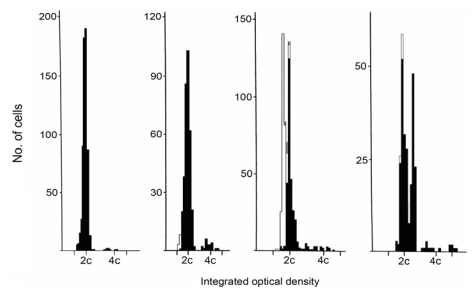
DNA ploidy distribution graphs for nuclei of tumour tissues. A; typical diploid display for the UK renal cell carcinoma (RCC) providing ‘control’ data for that of three Krogh tumour tissues. B; main mass of Krogh RCC (diploid), C; Krogh tumour extending into renal vein (diploid), D; Krogh distant aneuploid metastasis (hyperdiploid). Ploidy chart nuclear codes: black = renal epithelial cells, white = internal control lymphocytes, shaded = internal control fibroblasts.

**Figure 2:**
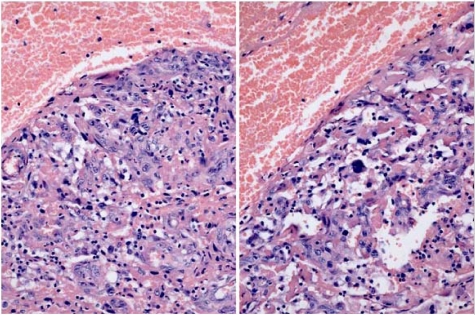
H & E stained histopathology of RCC extending into the renal vein, showing occasional pleomorphic nuclei.

**Figure 3:**
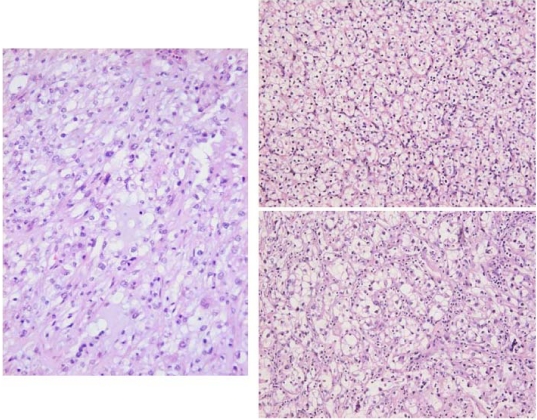
H & E stained histopathology of RCC. Left; UK ‘control’ tissue. Right above; Krogh’s typical clear cell renal tumour. Right below; disorganised aneuploid tissue of the distant metastasis. Synchronised magnification from a ×20 objective.

DNA ploidy distribution in Palle Krogh’s RCC, and in the control RCC, was diploid in both regions analysed (Figure 1 A, B). DNA in nuclei of a distant metastasis had an aneuploid distribution, with one main hyperdiploid peak and only occasional nuclei of higher ploidy (Figure 1D). The primary clear cell tumour (Figure 3B) had extended into the renal vein; this tissue also had a diploid distribution pattern (Figure 1C) matching the mass of tumour at the renal pole. However, a histological preparation of a serial section adjacent to those taken for ploidy analysis revealed occasional pleomorphic nuclei (Figure 2) near to the venous lumen. Whereas their ploidy status is unknown, it is possible that aneuploidisation had already commenced in this part of the primary tumour, from which metastatic dissemination might readily have occurred. 

**Figure 4:**
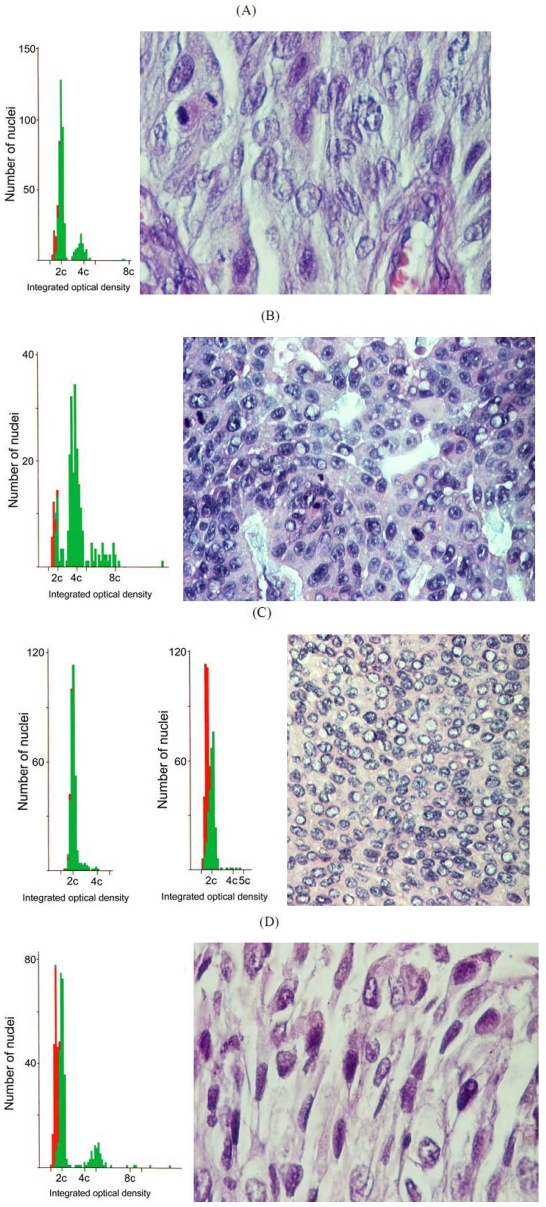
Matched DNA ploidy distribution and H & E stained histopathology of four Romanian transitional cell carcinomas (TCC) from upper urinary tract of subjects diagnosed with BEN. A, B and D: aneuploid tumours of cases 1, 2 and 4, respectively. C: Typical diploid data of a ‘control’ tumour from skin, adjacent to a similar pattern in a diploid TCC with matching histopathology (case 3). Notably, a hyperdiploid population of nuclei, as in the Krogh distant metastasis, is absent in all cases. Magnifications: A,D x 400 ; B,C x 200. Ploidy chart nuclear codes: green = renal epithelial cells, red = internal control lymphocytes, blue= internal control fibroblasts.

DNA ploidy in one of the Balkan TCC was diploid (Figure 4C; case 3), as was the skin tumour control (Figure 4C). However, the other three TCC were markedly aneuploid (Figure 4A, B, D; cases 1, 2, 4) although none of these distributions contained a significant hyperdiploid component. Case 1 aneuploidy was mainly hypertriploid, but in cases 2 and 4 aneuploidy extended considerably beyond tetraploid. The factors of tumour size and grading and association with pyelonephritis in the 4 tumours did not correlate with the degree of aneuploidisation. There was also considerable variation in the histopathology among the four Balkan TCC (Figure 4A-D).

## 3. Discussion

### 3.1. The renal cancer cases

The Krogh case study focuses on RCC developing silently in a Caucasian male in a developed Western European environment during middle age. The morbidity provoking surgical intervention occurred only when the primary tumour had already become rather large. However, although archived renal tumour had a diploid DNA distribution in the analysed tissue, subsequent survival for only two years after radical nephrectomy pointed to some further clonal selection for metastasis, prior to nephrectomy, as evidenced by the aneuploidy of a metastatic carcinoma discovered subsequently. Unfortunately, metastatic tissue from lung at the terminal stage had not been archived. However, it is tantalising to contemplate whether occasional nuclear instability in tumour invading the renal vein had already been a crucial step spawning aggressive metastasis to distant organs.

The picture fits closely into the current pattern exhibited by West European cases of RCC as shown in a recent publication on clinical and molecular aspects of the disease [25]. The case also conforms to recent surveys of DNA ploidy distribution in RCC cases coming to radical nephrectomy, with aneuploidisation discovered in some part of the primary tumour correlating with tumour classification, so that a switch from diploidy to aneuploidy is a feature developing late within a RCC [26,27]. Specifically, the significant hyperdiploid component in the Krogh metastatic tumour corresponds to the ploidy distribution illustrated as within the typical range for clear cell RCC [27]. The present ploidy analysis of both primary tumour and distant metastasis is possibly the first individual sequence reported for human RCC. For human renal tumours, ploidy analysis of up to five regions of the tissue is desirable for a reliable measure, but in many of the rat renal tumours attributed to OTA [21] analysis was made on all, or a large proportion of, the tumour. Pinto *et al*. [25] stated ‘patients presenting aneuploid tumours are estimated to be 15.6 times more likely to die of the disease than are those with diploid tumours’. The clear sequence from diploid primary tumour to aneuploid secondary tumour in the Krogh case is illustrative of the aneuploidisation principle, and it is tantalising to consider whether renal vein invasion had been involved in this process. In retrospect, a stage of T4N0M1 could be ascribed to the primary renal tumour, consistent with a poor actual outcome and particularly on account also of the venous invasion [28].

The mechanisms of initiation and proliferation of human RCC remain generally obscure. Some predisposing factors such as analgesic abuse (notably phenacetin) are recognised, but otherwise the disease seems just to be linked non-specifically to societies with an affluent lifestyle [29]. That specific DNA damage is necessary for the initial adenoma lesion, and again for differentiation into metastasising carcinoma, has justified study of the rare familial form of RCC, involving homozygous occurrence of the von Hippel Lindau recessive gene mutation, as an interesting putative model. However, study of early stages of human RCC is extremely difficult. Hence in the Krogh case, the close professional involvement in experimental ochratoxicosis A in farm and laboratory animals at a time when the possible human risk, as then perceived, could not have been fully appreciated, might have allowed unwise and un-natural exposure to the toxin. The pathological outcome might therefore be a pointer to OTA as a human carcinogen. For this reason, in the absence of any human cases in which OTA has been shown to cause any morbidity, several putative mechanisms have been proposed to account for carcinogenesis in the specific renal tumours that have been produced experimentally in male rats by OTA [30,31,32,33]. The relative contribution of these remains unclear while official uncertainty about the genotoxicity of OTA as a chemical carcinogen persists [34]. 

However, the present finding of rather limited aneuploidisation (hyperdiploid) only in the Krogh metastatic tumour does not fit comfortably with the marked nuclear instability implied from the extreme aneuploidy (up to ~32-ploid) in primary rat renal carcinomas caused by OTA [21] and the case can not provide evidence supporting the IARC classification of OTA as a potential human carcinogen [35]. In contrast, the extreme aneuploidy found in the TCC of most of the Romanian BEN patients, matches that found in rat OTA tumours [21] quite closely. Human tumours initially develop with minimal genetic damage, as revealed by the DNA ploidy measurement, but only develop nuclear instability at the stage at which they metastasise. OTA tumours in rats develop marked nuclear instability quite early, often *in situ* and certainly before metastasis, and develop multiple aneuploid stem lines and aneuploid cells early during proliferation of the mass. The Krogh RCC showed no evidence of this until metastasis and thus this human type is unlikely to be due to OTA. Wider study of nuclear instability in TCC of BEN patients from across the Balkans would assist in consolidating the apparent contrasting situation in RCC and TCC. The technique used here is particularly designed to enable measurements on wax-embedded archival tissue. 

### 3.2. Balkan endemic nephropathy: The ochratoxin A hypothesis

The aetiology of the insidious bilateral renal atrophy of BEN, and the TCC with which it is frequently associated, remains at most uncertain, as is also the extent to which the two disparate pathologies of atrophy and proliferation have a common cause. Krogh’s perception of OTA’s involvement concerned just the very slow human nephropathy, but was based on general extrapolation from the relatively more rapid Danish mycotoxic nephropathy in bacon pig production. However, there has always been the uncomfortable juxtaposition of the marked renal hypertrophy in pigs (also associated with impaired live-weight gain) with the progressive silent renal atrophy in BEN, which are not easy to reconcile through a common pathological mechanism [36]. A more plausible role for OTA might concern the TCC in BEN patients, because of a presumed greater availability of unmetabolised OTA passing through partially dysfunctional fibrotic kidney and thus having access to the transitional cell epithelia of the renal pelvis and ureter. Increased OTA concentration has also been detected in sera of BEN patients from Croatia, compared to controls [37], supporting the idea of a dysfunctional kidney unable to clear the mycotoxin efficiently from the body. Moreover, the principal DNA adduct attributed to covalent binding with OTA was detected in three Balkan renal pelvis tumours, but also in bladder tumours [38,39]. However, it is important to know whether evidence of genuine OTA/DNA adducts in tumour tissue reflects their tumour-causing role for the mycotoxin, or shows an attractive proliferating depot in a morbid ageing subject where traces of circulating OTA can accumulate in malignant tumour. The evidence could just represent genetic damage that was unrepairable by malignant tumour. Study of tumours in non-urological tissues of BEN subjects could reveal data to clarify any specificity to urological tissue. In any case, in establishing an aetiological connection of OTA with all or part of the pathologies of BEN it would be vital to demonstrate consistent correlation of evidence of toxin exposure with disease development; correlation only in a proportion of cases would not suffice for satisfactory proof.

Upper tract urothelial tumours [40] occurring in some BEN patients are all transitional cell neoplasms irrespective of their close proximity to the kidney in the renal pelvis. In contrast, the most potent expression of experimental renal cancer specifically by OTA is in the male rat, but tumours are all closely associated with the renal parenchyma and are found in ageing animals, even if OTA exposure occurs only during the first third of a normal lifespan.

Extensive literature on analysis for OTA in food commodities and human diets, and also human blood, records the widespread occurrence of traces of the toxin, but no notable pattern of exceptional geographic foci [41,42]. However, a dietary study in 16 young healthy adults (gender not specified) in two Bulgarian hyperendemic villages found consistent exposure to OTA [43], but in two subjects the amounts were close to or slightly exceeded the current provisional tolerable weekly intake (100 ng/ kg body weight) recommended at that time by JECFA. By definition, this may not impose a health risk, but these individuals (if identified) may be particularly valuable for long-term assessment of OTA toxicology. However, another survey in the same region had found hardly different mean daily intake of OTA (1.21 µg and 1.03 µg, respectively) in BEN households versus households in non-BEN villages [44]. 

**Figure 5:**
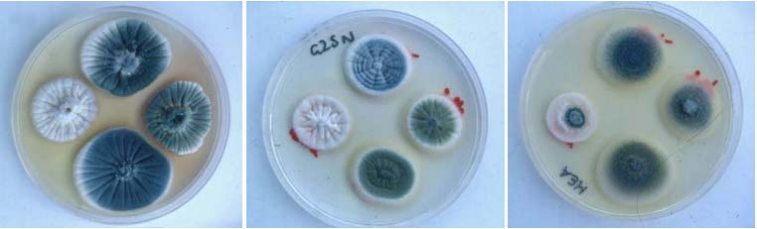
Fungi isolated from maize (containing ochratoxin A) in a BEN household in Gorno Peshtene, Bulgaria cultured for taxonomic identification according to Pitt [45] on Czapek-yeast agar (left), G25N agar (centre) and malt extract agar (right) for 7 days at 25 ºC. Within each plate: *P. verucosum* (left), *P. griseofulvum* (above), *P*. *solitum* (below), *P. viridicatum* (right). The latter three isolates were ochratoxinogenic [14].

Even the sources of the OTA detected in some BEN areas are still unclear. The dominant OTA-producing fungus in Northern Europe is *P. verrucosum*, and of more tropical areas is *A. ochraceus* and the black-spored species *A. niger* and *A. carbonarius.* Studies on food-borne moulds in the specific houses in the Croatian village of Kaniza [12] revealed none of the above. One isolate of *P. variabile* showed blue fluorescence in agar culture, but this was not due to OTA. A more detailed study in 20 BEN households across five Bulgarian hyperendemic villages [13] found one example of *A. ochraceus*, with a weak expression of OTA biosynthesis, and two atypical forms of *P. verrucosum* which did not produce OTA. However, two Penicillia (Figure 5) from maize, *P. griseofulvum* (IMI 351303) and an atypical *P. viridicatum*, produced OTA in laboratory culture on shredded wheat (1-2 ppb). Also, an isolate (Figure 5) assigned as another atypically-ochratoxinogenic species (*P. solitum* (IMI 351303), supported significant OTA productivity (1.3 ppm) in shredded wheat. These fungi had been isolated from the hyperendemic village of Gorno Peshtene, also included in the study by Vrabcheva *et al*. [43]. Subsequent study of low-grade local cereals, distant from the Bulgarian BEN endemic area and used for feed in pig production units where mycotoxic porcine nephropathy occurred, revealed no obvious ochratoxinogenic fungi [15]. Clearly there has been considerable under-investment in understanding the mycological epidemiological aspect of putative mycotoxin involvement in BEN.

## 4. Conclusions

Potential for application of the present technique for DNA ploidy measurement in Balkan TCC, archived in wax blocks or preserved in formalin, has been demonstrated in this pilot study and could add valuable information on the contrasting extent of nuclear instability in primary TCC and RCC. However, further analysis would require a well-funded and dedicated programme. It should be remembered that the precise aetiological relationship between the silent bilateral renal atrophy of BEN and the upper urinary tract TCC that arise in some cases remains unclear. To define the extent of nuclear instability across the resource of tumours archived within the Balkan countries could add fundamental information to which mode(s) of mutagenic mechanism of putative toxicants (including OTA) in animal models might be compared for a possible fit.

## 5. Experimental Section

### 5.1. Ploidy analysis

DNA ploidy distribution analysis was performed as previously described [22] and applied recently to rat renal tumours [21]. Briefly, several 50 µm sections were cut from selected areas of blocks, dewaxed in xylene, rehydrated and nuclei extracted using protease type XXIV (Sigma Chemical Co., Poole, UK) [23]. Nuclear monolayers were prepared in a cytospin 4 (Thermo Shandon, UK) and stained by the Feulgen-Schiff method and analysed on a Fairfield image-based ploidy analyzer (Medical Solutions, UK). Monolayers were analysed using the automated scanning option. Diagnostic criteria for ploidy status were as previously published for this system. A sample was classified as diploid if only one G0/G1 (2c) peak was present, or if the number of nuclei in the G2 (4c) peak did not exceed 1% of the total number of epithelial cells. A lesion was defined as aneuploid if non-euploid peaks were present, or if the number of nuclei with DNA content greater than 5c exceeded 1%.

### 5.2. Tissues relating to renal cell carcinoma

**Figure 6:**
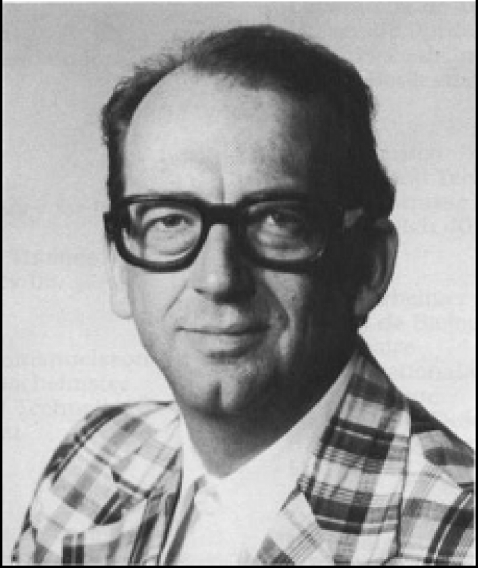
Prof. Palle Krogh (1935-1990).

Tumour details for Palle Krogh (Figure 6): In June 1988 (age 53) CT-scanning revealed an 8 cm tumour in the lower part of the right kidney. Needle aspiration showed cells suspicious of renal cell carcinoma (RCC). One month later, radical nephrectomy removed a carcinoma (9 × 8 × 8 cm) located at the lower pole. The cut surface had areas of necrosis and haemorrhage. The tumour did not obviously extend beyond the capsule, but did extend into the renal vein. A tumour thrombus (10 × 5 × 5 mm) was removed from the inferior vena cava. Microscopically, it was a typical RCC of clear cell type, moderately differentiated. Tumour tissue with similar morphology was found in the renal vein and in the inferior vena cava. The primary renal cell tumour at operation was staged as T3bN0M0. In September 1989 a solitary metastasis of a clear cell carcinoma was removed from soft tissue in the right femoral region. Notably, recent MRI illustration of a femoral metastatic deposit is shown in [24]. In February 1990 biopsies from the right main bronchus, lower right bronchus and at the bifurcation showed metastasis from RCC; histopathology was of the clear cell type.

Control male: Currently resident in England, age 56 at radical left nephrectomy. The tumourous kidney (300 g) had a partially necrotic and haemorrhagic tumour at the upper pole of the kidney (11 × 9 × 6 cm). There were multiple external nodules (largest 1.5 cm in diameter) in perirenal fat. Microscopically, the tumour was a moderately differentiated RCC with areas of haemorrhage and necrosis; the tumour infiltrated through the capsule into perirenal fat. Areas showed lymphovascular invasion, but renal vein and large arteries were free of tumour. Pathological grade was Fuhrman’s grade 2 and Robens stage at least 2. Additionally, bladder mucosa had a well-differentiated papilliary transitional cell carcinoma, pathological grade G1pTa. 

### 5.3. Romanian transitional cell tumours (TCC) from female BEN patients (2002-2004):

Case 1; 75 years. Gross: Left kidney, tumour located in the renal hilum, 3 × 1.5 × 1.5 cm, exophytic aspect, friable. Microscopy: TCC, papillary, moderately differentiated G2. Chronic pyelonephritis in the adjacent parenchyma. Proximal ureter and bladder wall not affected.

Case 2; 70 years. Gross: Dorso-lumbar wall tumour, infiltrating the fat, muscle and upper dermis, 3 × 3.8 cm, solid, high consistency. Microscopy: TCC, moderately differentiated G2, associated to poorly differentiated adenocarcinoma. 

Case 3; 65 years. Gross: Left kidney, tumour of pelvic origin, 7 × 6.5 cm, papillary aspect, friable, necrotic-haemorrhagic. Microscopy: TCC, moderately/poorly differentiated G2/3, with foci of squamous differentiation, tumor necrosis, hyalinization, calcifications and vascular invasion. Chronic pyelonephritis in the adjacent parenchyma. No tumour invasion into proximal or distal ureter.

Case 4; 74 years. Gross: Left kidney, tumour of pelvic origin, One dilated calyx containing a slightly raised tumour, 0.2 × 1 × 1.5 cm. Microscopy: TCC, poorly differentiated G3, infiltrating the submucosa. Chronic pyelonephritis in the adjacent parenchyma. Tumour invasion of ureter as small, multiple islands of the same cancer type, infiltrating the whole ureter wall.

Control female: 69 years in 2001. Romanian benign papillomatous skin tumour, not associated with BEN
